# Supplementary material for: Tropical cyclones act to intensify El Niño
Source: Nat Commun. 2019 Aug 22;10:3793. doi: 10.1038/s41467-019-11720-w (PMC6706434; doi:10.1038/s41467-019-11720-w)
Supplement: Supplementary file 1 — Supplementary Information [file 41467_2019_11720_MOESM1_ESM.pdf]

Supplementary Information

Tropical cyclones act to intensify El Niño

By Wang et al.

Supplementary Figures

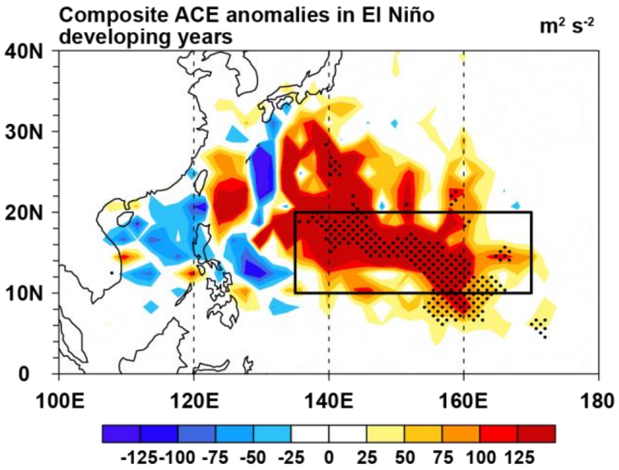

Supplementary Figure 1 Composite of the accumulated cyclone energy (ACE) anomalies (shading, m^2 s^{-2}) over the western North Pacific during the El Niño developing years (all months) from 1970 to 2016. An El Niño developing year is defined when the above-moderate El Niño events (including moderate events) develops from weak to strong. The stippled regions indicate significance above the 99% confidence level using Student's *t*-test. The black rectangle denotes the selected ACE region (10°–20°N, 135°–170°E).

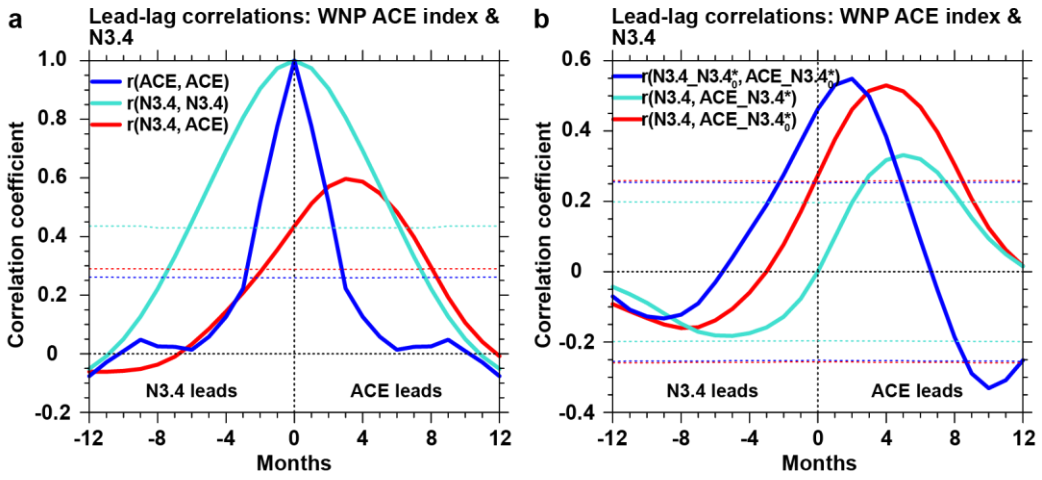

Supplementary Figure 2 Lead–lag correlations between the accumulated cyclone energy (ACE) anomalies over the western North Pacific (10°–20°N, 135°–170°E; WNP) and the running 3-month mean SST anomaly for the Niño 3.4 region (5°N–5°S, 120°–170°W) (N3.4, an ENSO index) together with their autocorrelations in the period 1970–2016. **a**, Lead–lag correlation and autocorrelations between original series. The red, turquoise and blue dashed lines indicate significance at the 99% confidence level related to lead–lag correlations between the WNP ACE and N3.4 indices, and autocorrelations of the N3.4 and WNP ACE indices via Student's *t*-test using the effective number of degrees of freedom, respectively. **b**, Lead–lag correlations between the processed series. N3.4_N3.4$_0^*$ (ACE_N3.4$_0^*$) indicates the N3.4 (WNP ACE) not associated with the preceding (3 months earlier) N3.4. ACE_N3.4* indicates the WNP ACE index not associated with the simultaneous N3.4. The red, turquoise and blue dashed lines indicate significance at the 99% confidence level related to lead–lag correlations between N3.4 and ACE_N3.4$_0^*$, N3.4 and ACE_N3.4*, N3.4_N3.4$_0^*$ and ACE_N3.4$_0^*$ via Student's *t*-test using the effective number of degrees of freedom, respectively.

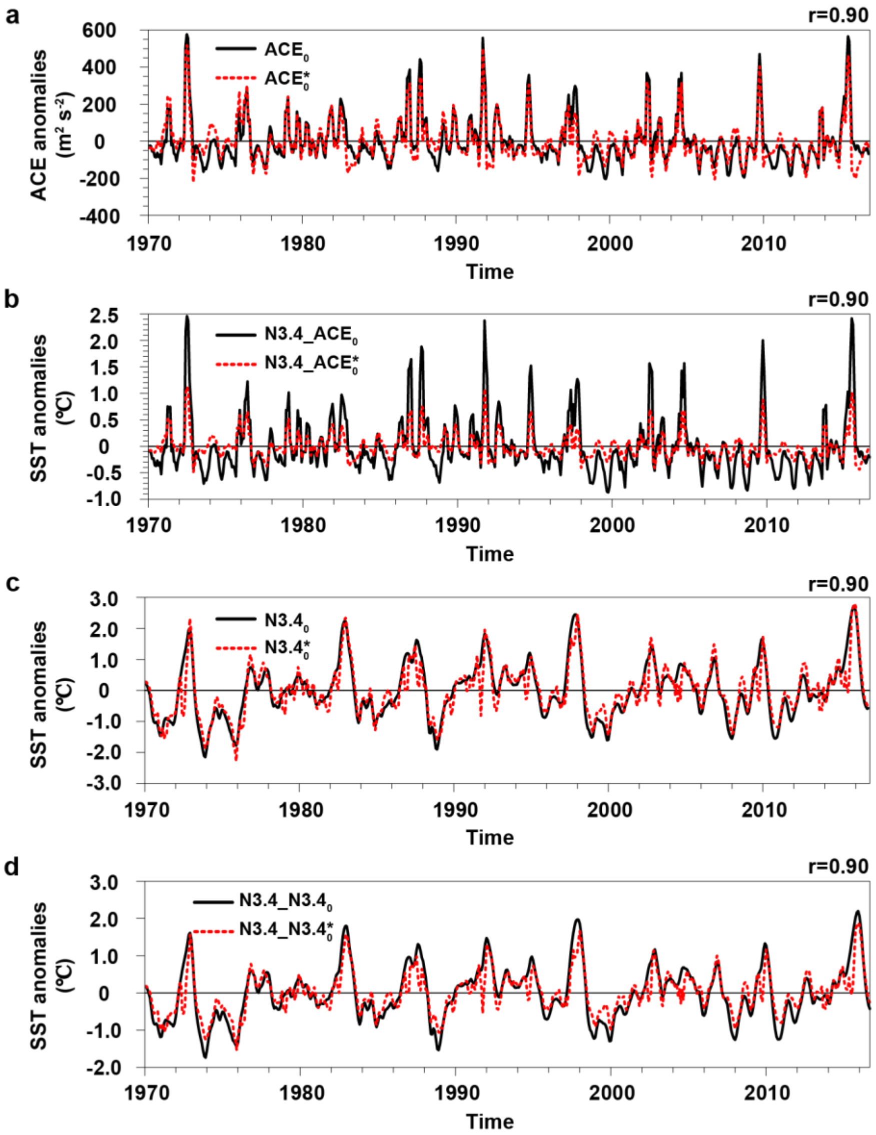

Supplementary Figure 3 Time series of the preceding (3 months earlier) accumulated cyclone energy (ACE) anomalies (m^2 s^{-2}) over the western North Pacific (10°–20°N, 135°–170°E; WNP) and the running 3-month mean SST anomaly for the Niño 3.4 region (5°N–5°S, 120°–170°W) (N3.4, an ENSO index, °C), and regressions onto the N3.4 from 1970 to 2016. **a**, Preceding ACE. ACE_0 denotes the original series, and ACE_0^* indicates the series not associated with the preceding N3.4. **b**, Regression on the N3.4. N3.4_ACE_0 denotes the regression of the preceding ACE index on the N3.4, and N3.4_ACE_0^* indicates the regression of the preceding ACE index not associated with the preceding N3.4 on the N3.4. **c**, Preceding N3.4. $N3.4_0$ denotes the original series, and $N3.4_0^*$ indicates the series not associated with the preceding ACE index. **d**, Regression on the N3.4. N3.4_$N3.4_0$ denotes the regression of the preceding N3.4 on the N3.4, and N3.4_$N3.4_0^*$ indicates the regression of the preceding N3.4 signal not associated with the preceding ACE on the N3.4. Here, *r* is the correlation coefficient.

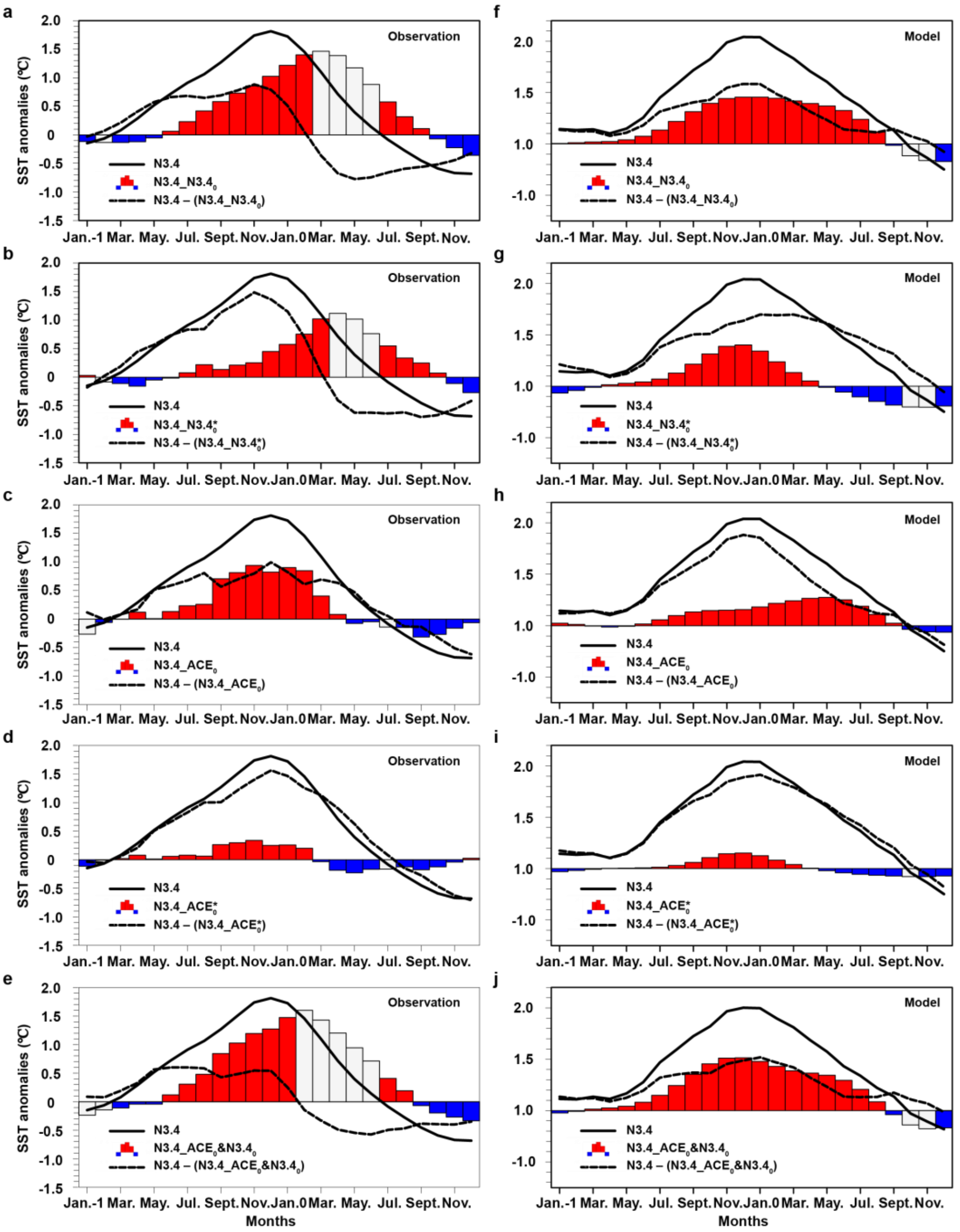

Supplementary Figure 4 Composite time series of the running 3-month mean SST anomaly for the Niño 3.4 region (5°N–5°S, 120°–170°W) (N3.4, an ENSO index, °C) for the original (solid line) and preceding-contributor-independent (dashed line) above-moderate El Niño events (including moderate events), as well as the explained percentage (%) of Niño-3.4 intensity (bars) obtained using the preceding contributors' signal. ACE_0&$N3.4_0$ represents the combined contribution of the preceding the accumulated cyclone energy (ACE) over the western North Pacific (10°–20°N, 135°–170°E; WNP) and N3.4; $N3.4_0$ the preceding N3.4; ACE_0 the preceding WNP ACE; $N3.4_0^*$ the preceding ACE-independent N3.4 (i.e., the preceding N3.4 after removing WNP ACE); and ACE_0^* the preceding N3.4-independent ACE index

(i.e., the preceding WNP ACE after removing N3.4). **a–e**, Observations. **f–j**, As in **a–e**, but for the model results. Red (blue) bars represent the positive (negative) explained percentage of the preceding signals to the N3.4, and whitesmoke bars indicate the explained percentage of the preceding signals to the N3.4 with the sign of the N3.4 changing. Jan.-1 and Jan.0 represent the January in last year and the year concurring with El Niño, respectively.

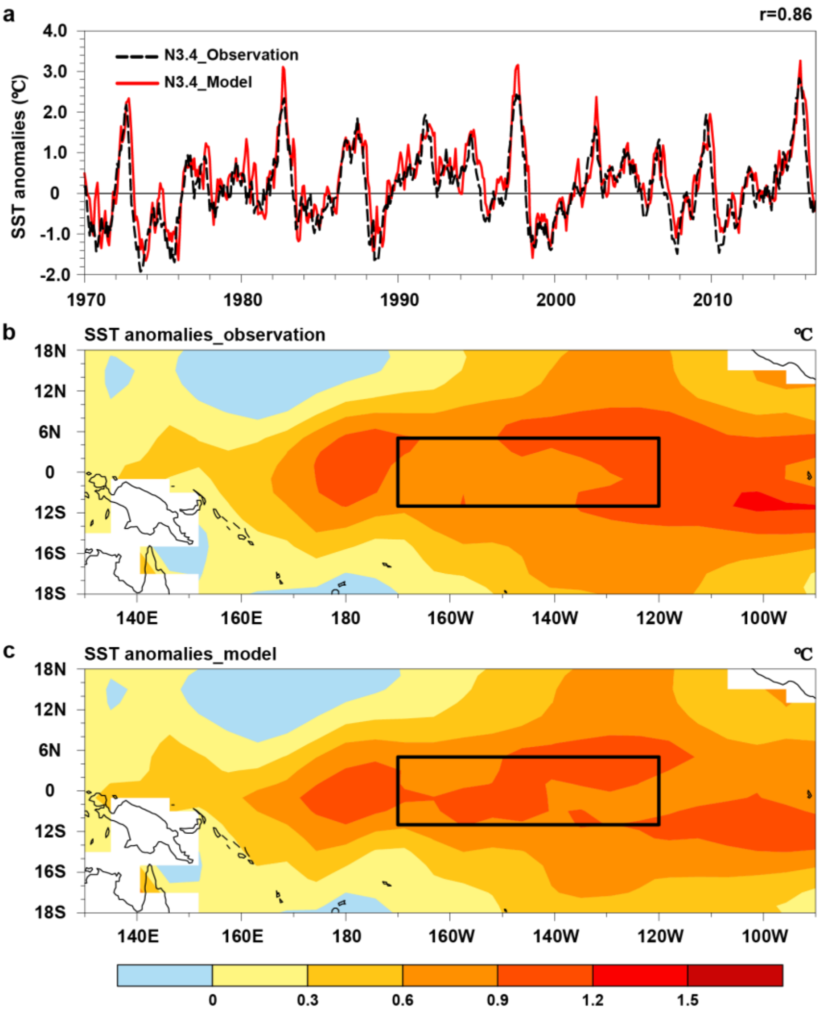

Supplementary Figure 5 Time series of the succeeding (3 months later) running 3-month mean SST anomaly for the Niño 3.4 region (5°N–5°S, 120°–170°W) (N3.4, an ENSO index, °C) from 1970 to 2016 and spatial distributions of sea-surface temperature anomalies (SST, shading, °C) during the El Niño developing years. a, Time series of the observed (red line) and predicted (black line) N3.4 from an intermediate-complexity coupled ocean–atmosphere model[10,11]. *r* is the correlation coefficient; **b**, Observations; **c**, Predictions. The black rectangle denotes the selected the Niño-3.4 region (5°S–5°N, 120°–170°W). An El Niño developing year is defined when the above-moderate El Niño events (including moderate events) develops from weak to strong.

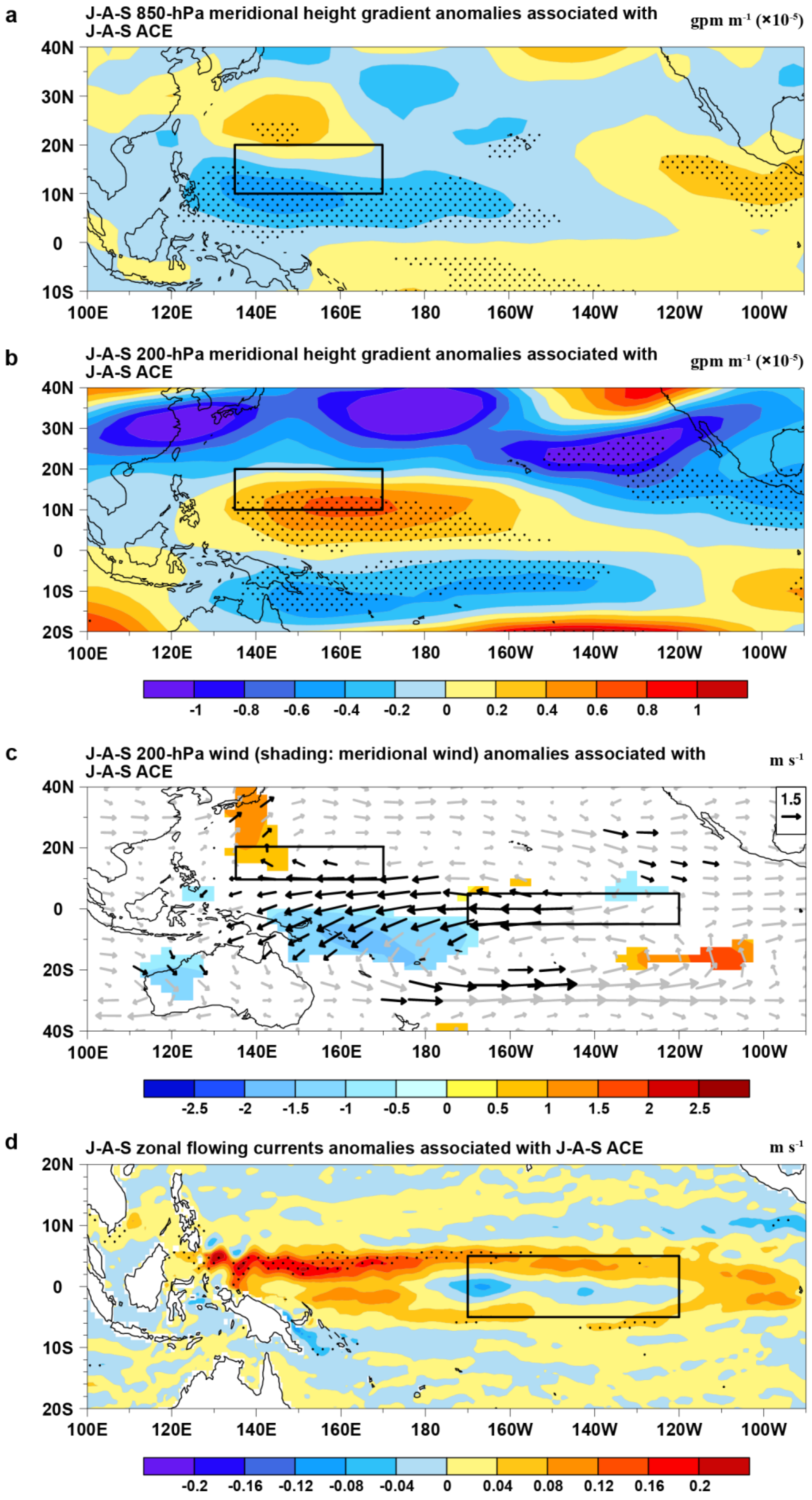
a
J-A-S 850-hPa meridional height gradient anomalies associated with J-A-S ACE
gpm m-1 (×10-5)
40N
30N
20N
10N
0
10S
100E
120E
140E
160E
180
160W
140W
120W
100W
b
J-A-S 200-hPa meridional height gradient anomalies associated with J-A-S ACE
gpm m-1 (×10-5)
20S
-1
-0.8
-0.6
-0.4
-0.2
0
0.2
0.4
0.6
0.8
1
c
J-A-S 200-hPa wind (shading: meridional wind) anomalies associated with J-A-S ACE
m s-1
1.5
40S
-2.5
-2
-1.5
-1
-0.5
0.5
1
1.5
2
2.5
d
J-A-S zonal flowing currents anomalies associated with J-A-S ACE
m s-1
-0.2
-0.16
-0.12
-0.08
-0.04
0.04
0.08
0.12
0.16
0.2

Supplementary Figure 6 Composite fields for each month from July to September associated with the simultaneous accumulated cyclone energy (ACE) anomalies over the western North Pacific (10°–20°N, 135°–170°E; WNP) during El Niño developing years (1970–2016). **a**, 850-hPa meridional geopotential height gradient anomalies ($\times10^{-5}$, gpm m^{-1}). **b**, 200-hPa meridional geopotential height gradient anomalies ($\times10^{-5}$, gpm m^{-1}). **c**, Meridional (shading, m s^{-1}) and horizontal 200-hPa wind anomalies (vectors, m s^{-1}). **d**, Zonal flowing currents (m s^{-1}) at a depth of 5 m. Shading and black vectors in **c** as well as the stippled regions in **a**, **b,** and **d** indicate significance above the 95% confidence level using Student's *t*-test. The black rectangles denote the selected ACE region (10°–20°N, 135°–170°E) and the Niño-3.4 region (5°S–5°N, 120°–170°W). An El Niño developing year is defined when the above-moderate El Niño events (including moderate events) develops from weak to strong.

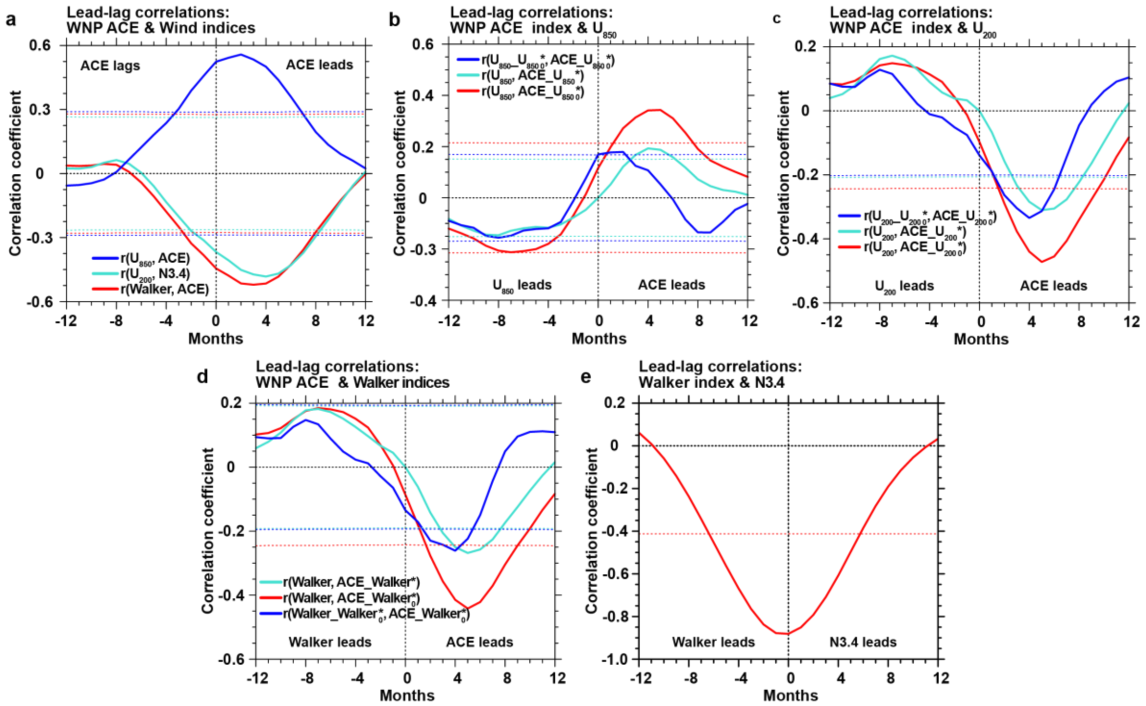

Supplementary Figure 7 Lead–lag correlations between the accumulated cyclone energy (ACE) anomalies over the western North Pacific (10°–20°N, 135°–170°E; WNP), wind (including the Walker index, zonal winds at 200 hPa (U_{200}) and 850 hPa (U_{850}), and the running 3-month mean SST anomaly for the Niño 3.4 region (5°N–5°S, 120°–170°W) (N3.4, an ENSO index) in the period 1970–2016. a, Lead–lag correlation between the WNP ACE and wind indices. The turquoise, red and blue lines indicate the 99% confidence levels related to lead–lag correlations between U_{200} and N3.4 indices, Walker and N3.4 indices, U_{850} and N3.4 indices via Student's *t*-test using the effective number of degrees of freedom, respectively. **b**, Lead-lag correlations between the processed ACE and U_{850} series. $U_{850}\_U_{850\,0}^{*}$ ($ACE\_U_{850\,0}^{*}$) indicates the U_{850} (WNP ACE index) not associated with the preceding (3 months earlier) U_{850}. $ACE\_U_{850}^{*}$ indicates the WNP ACE index not associated with the simultaneous U_{850}. The red, turquoise and blue dashed lines indicate significance at the 98% confidence level related to lead–lag correlations between U_{850} and $ACE\_U_{850\,0}^{*}$, U_{850} and $ACE\_U_{850}^{*}$, $U_{850}\_U_{850\,0}^{*}$ and $ACE\_U_{850\,0}^{*}$ via Student's *t*-test using the effective number of degrees of freedom, respectively. **c**, As in **b**, but for the processed ACE and U_{200} series. The red, turquoise and blue dashed lines indicate significance at the 99% confidence level. **d**, As in **c**, but for the processed ACE and Walker index series. **e**, As in **c**, but for the N3.4 and Walker indices.

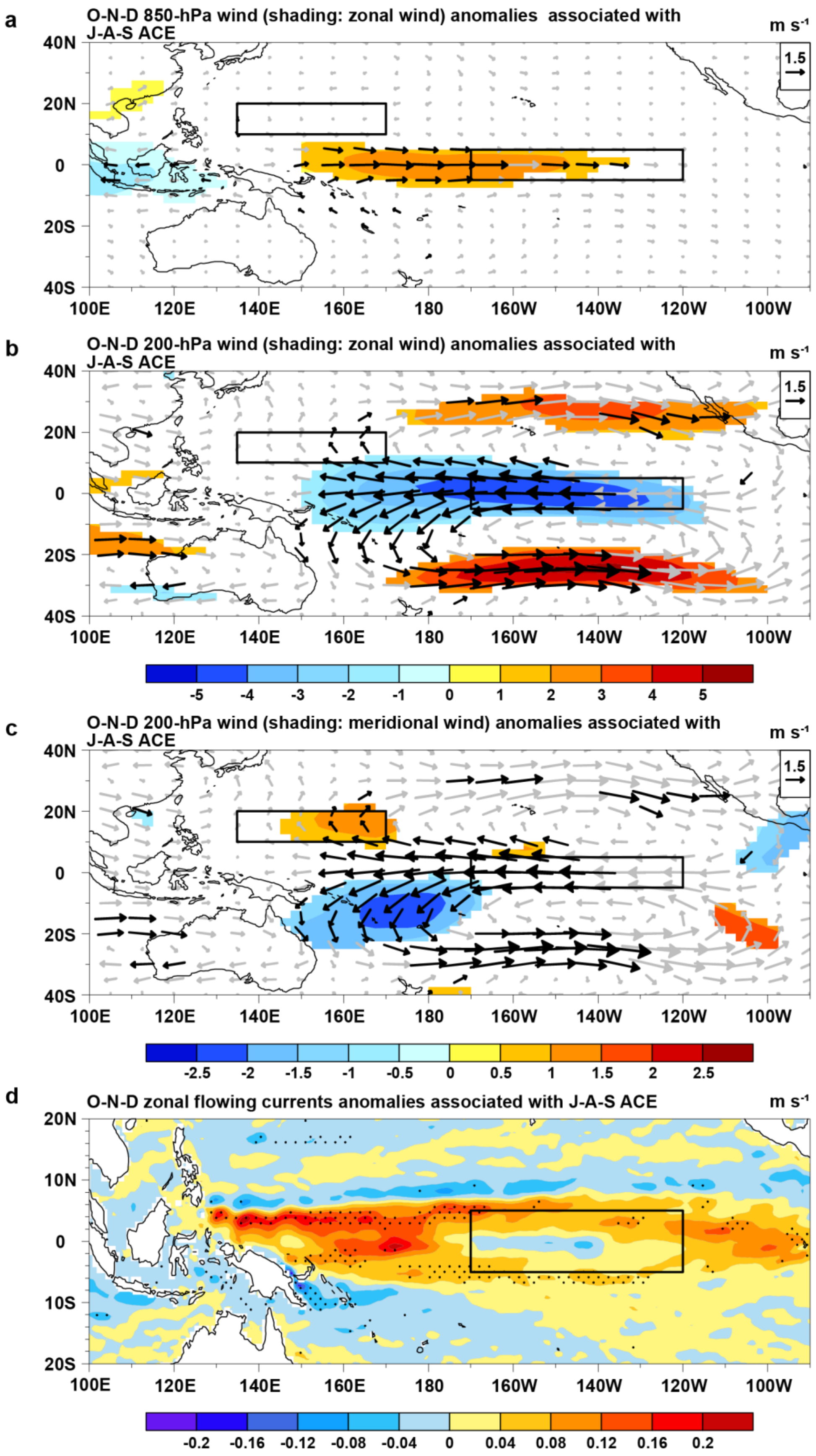

Supplementary Figure 8 Composite of fields in each month from October to December associated with the accumulated cyclone energy (ACE) anomalies over

the western North Pacific (10°–20°N, 135°–170°E; WNP) from July to September during El Niño developing years (1970–2016). a, Zonal and horizontal 850-hPa wind anomalies (m s^{-1}). b, As in a, but for 200 hPa. c. Meridional and horizontal 200-hPa wind anomalies (m s^{-1}). d, Zonal flowing currents (m s^{-1}) at a depth of 5 m in the Pacific. Shading and black vectors in a, b and c as well as the stippled regions in d indicate significance above the 95% confidence level using Student's *t*-test. The black rectangles denote the selected ACE region (10°–20°N, 135°–170°E) and the Niño-3.4 region (5°S–5°N, 120°–170°W). An El Niño developing year is defined when the above-moderate El Niño events (including moderate events) develops from weak to strong.

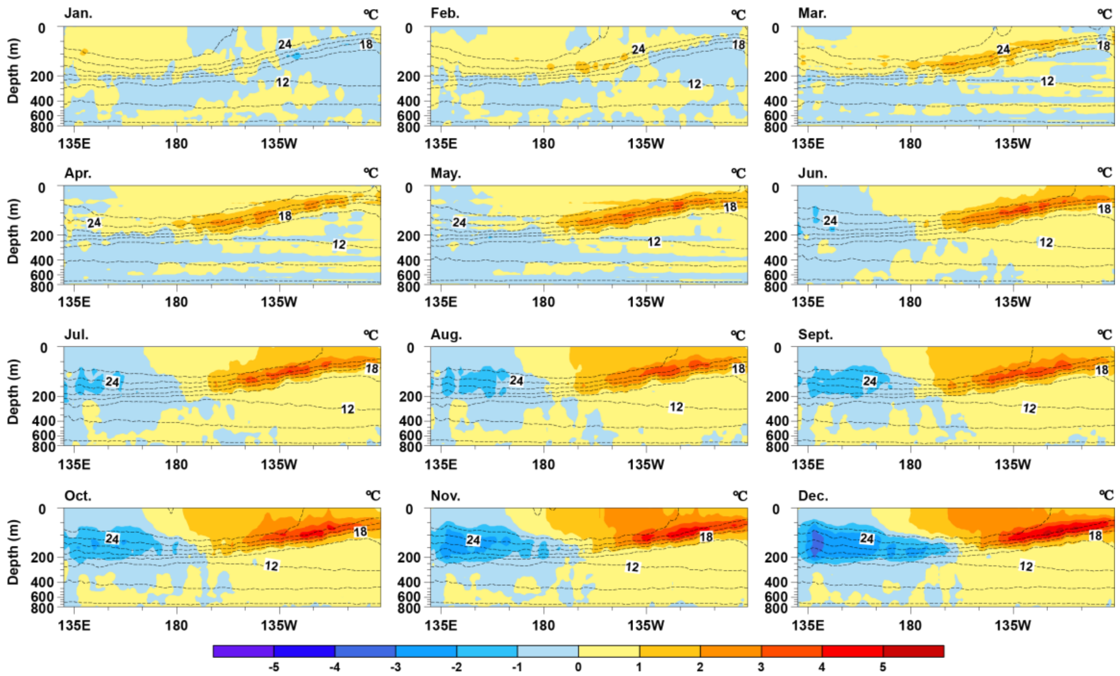

Supplementary Figure 9 Composite of the observational depth–zonal distributions of monthly equatorial potential temperature anomalies averaged between 5°S–5°N (shading; °C) during the El Niño developing years (2004–2016). An El Niño developing year is defined when the above-moderate El Niño events (including moderate events) develops from weak to strong. Dashed contours denote the isotherms of the potential temperature.

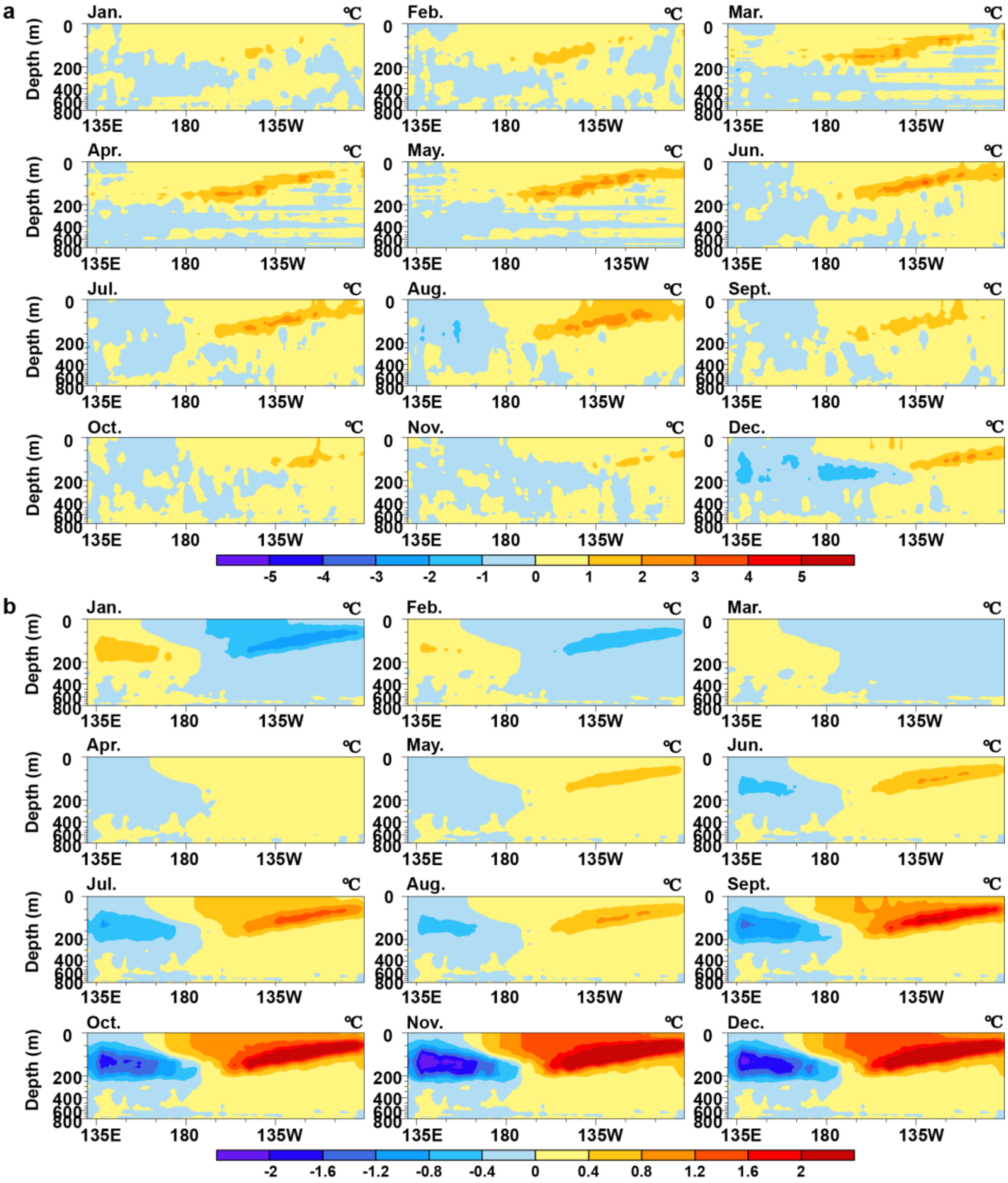

Supplementary Figure 10 Composite of depth–zonal distributions of monthly equatorial potential temperature anomalies averaged between 5°S–5°N (shading; °C) during the El Niño developing years (2004–2016). a, Distribution of monthly equatorial potential temperature anomalies after removing the proceeding (3 months earlier) WNP ACE. **b**, As in **a**, but for that relating to the proceeding WNP ACE. An El Niño developing year is defined when the above-moderate El Niño events (including moderate events) develops from weak to strong.

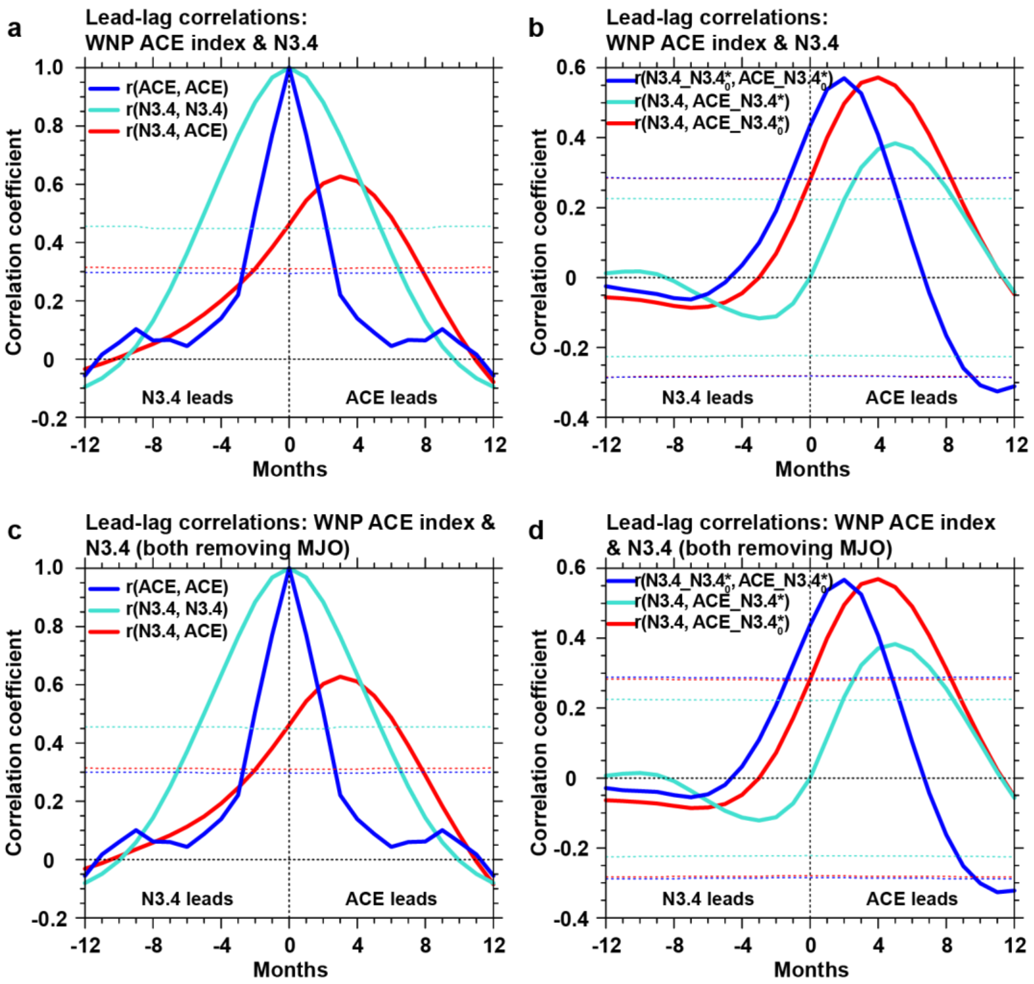

Supplementary Figure 11 Lead–lag correlations between the accumulated cyclone energy (ACE) anomalies over the western North Pacific (10°–20°N, 135°–170°E; WNP) and the running 3-month mean SST anomaly for the Niño 3.4 region (5°N–5°S, 120°–170°W) (N3.4, an ENSO index) together with their autocorrelations in the period 1979–2016. a–b As in Supplementary Figure 2, but for the ERA Interim dataset. **c–d**, as in **a–b**, but for the WNP ACE and N3.4 after removal of the Madden–Julian Oscillation (MJO) index.

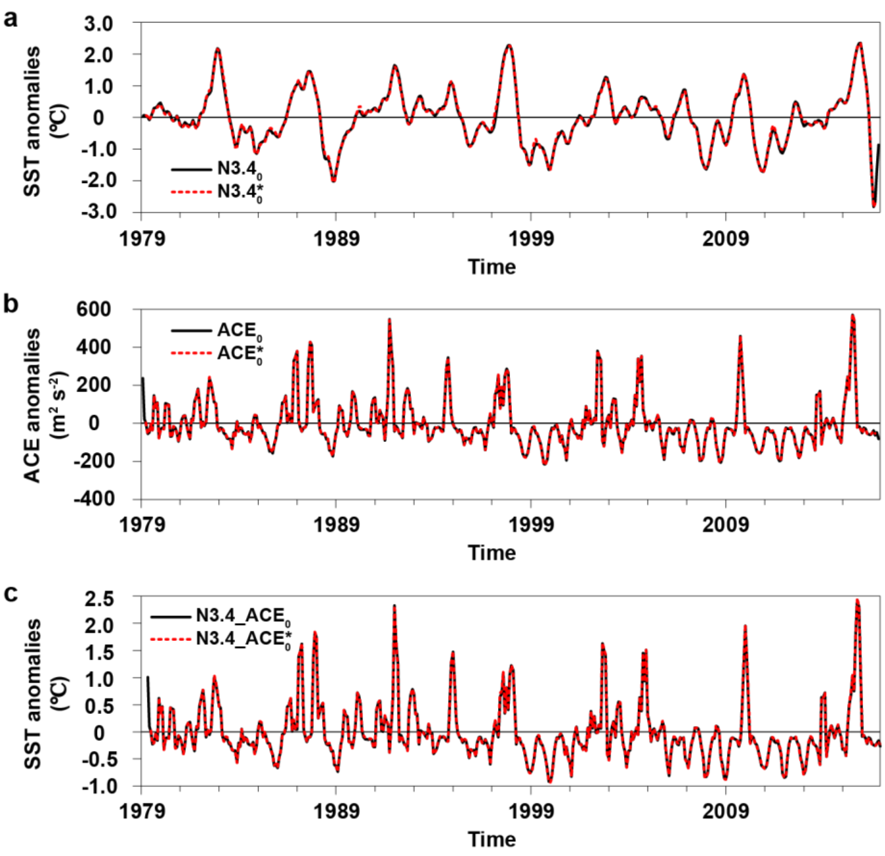

Supplementary Figure 12 Time series of the preceding (3 months earlier) accumulated cyclone energy (ACE) anomalies ($m^2 s^{-2}$) over the western North Pacific (10°–20°N, 135°–170°E; WNP) and the running 3-month mean SST anomaly for the Niño 3.4 region (5°N–5°S, 120°–170°W) (N3.4, an ENSO index, °C), and regressions onto the N3.4 from 1979 to 2016. **a**, Preceding N3.4. $N3.4_0$ denotes the original series, and $N3.4_0^*$ indicates the series not associated with the Madden–Julian Oscillation (MJO) index. **b**, Preceding ACE. ACE_0 denotes the original series, and ACE_0^* indicates the series not associated with the preceding MJO. **c**, Regression on the N3.4. $N3.4\_ACE_0$ denotes the regression of the preceding ACE index on the N3.4, and $N3.4\_ACE_0^*$ indicates the regression of the preceding ACE index not associated with the MJO on the N3.4.

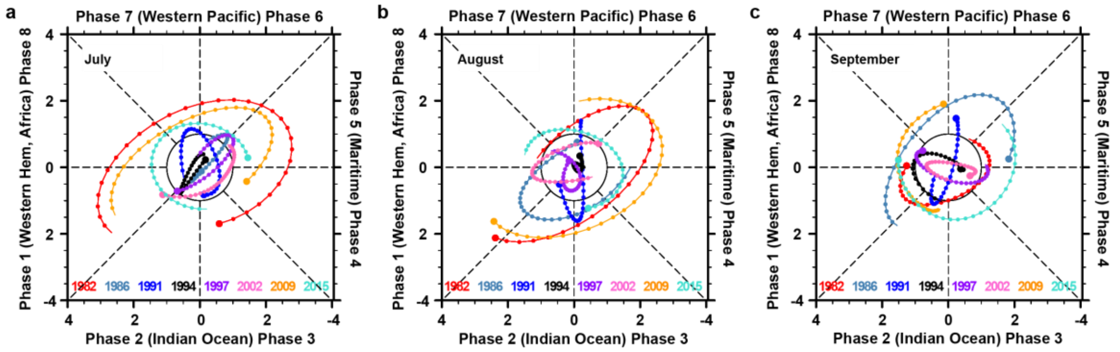

Supplementary Figure 13 Phase distribution of the daily Madden–Julian Oscillation index in each month from July to September during El Niño developing years in the period 1979–2016. a, July. **b**, August. **c**, September. Large dots indicate the first day of each month, small dots are one day, and lines are one year. An El Niño developing year is defined when the above-moderate El Niño events (including moderate events) develops from weak to strong.

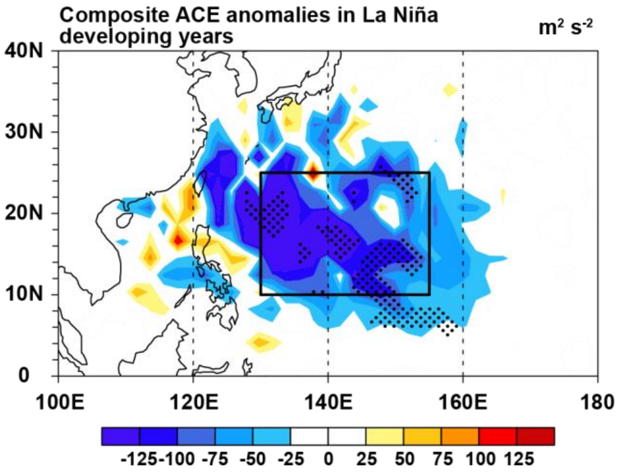

Supplementary Figure 14 Composite of the accumulated cyclone energy (ACE) anomalies (shading, m^2 s^{-2}) over the western North Pacific during the La Niña developing years (all months) from 1970 to 2016. The stippled regions indicate statistical significance above the 99% confidence level (Student's *t*-test). The black rectangle denotes the selected ACE region (10°–25°N, 130°–155°E). A La Niña developing year is defined when the above-moderate La Niña events (including moderate events) develops from weak to strong.

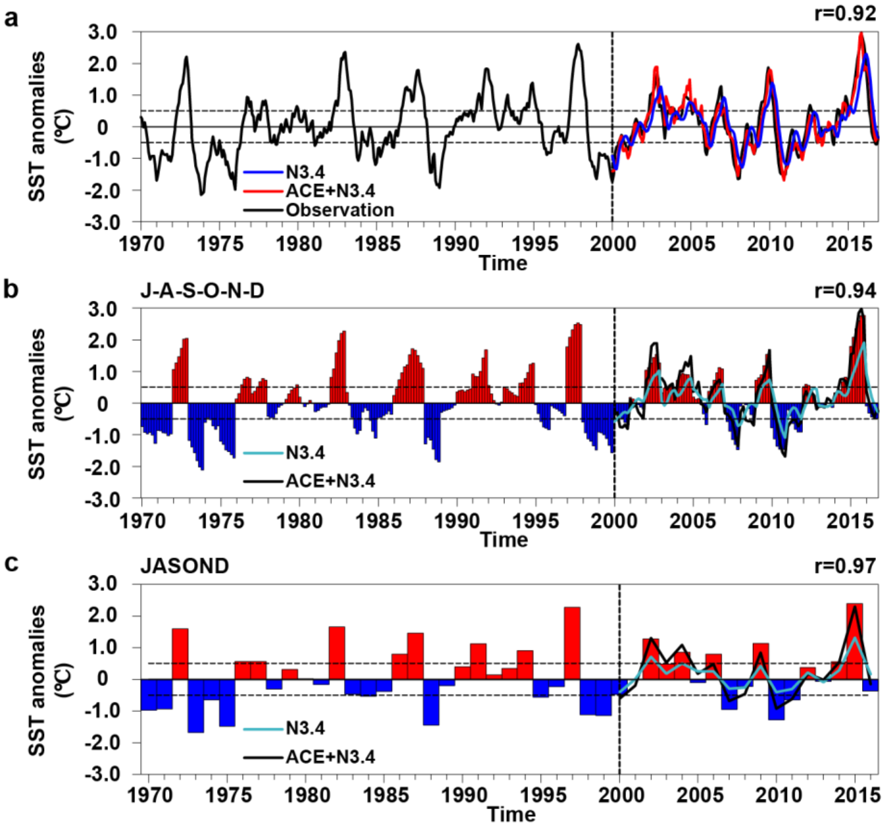

Supplementary Figure 15 Time series of the running 3-month mean SST anomaly for the Niño 3.4 region (5°N–5°S, 120°–170°W) (N3.4, an ENSO index, °C) for observations and predictions using the running holdout method. a, Time series of N3.4 for all months in the period 1970–2016. The two horizontal black dashed lines indicate 0.5°C and –0.5°C N3.4, and the vertical line divides the training and hindcasting periods. The black solid line indicates observations. The blue (red) solid line is the prediction from the N3.4 model (ACE+N3.4 model) for the period 2000–2016 and *r* is the correlation coefficient. **b**, Same as **a**, but for July–December (J–A–S–O–N–D). Bars denote observations and red (blue) indicates positive (negative) anomalies. Turquoise (black) solid line is the prediction from the N3.4 model (ACE+N3.4 model) in the period 2000–2016. **c**, Same as **b**, but for the mean value from July to December (JASOND).

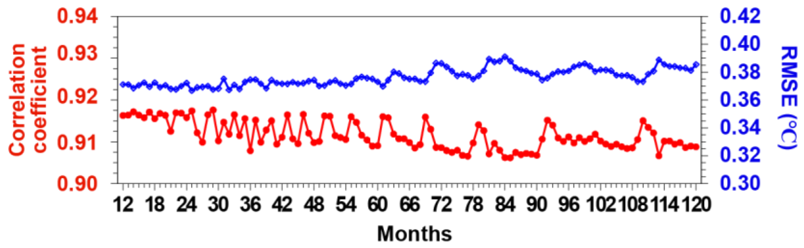

Supplementary Figure 16 Correlation coefficient and root mean square error (RMSE, °C) between the observed and predicted running 3-month mean SST anomaly for the Niño 3.4 region (5°N–5°S, 120°–170°W) (N3.4, an ENSO index), from ACE+N3.4 model using the method of Leave-P-out Cross-Validation.

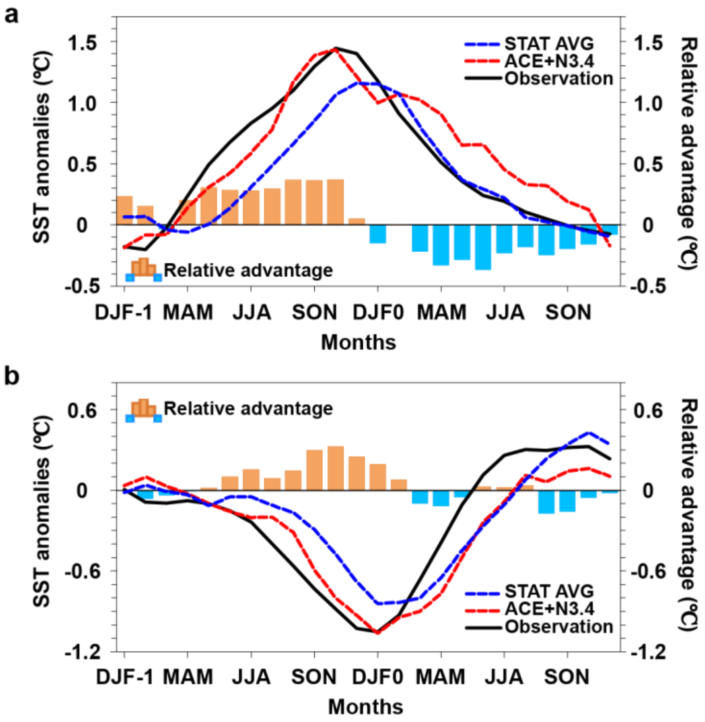

Supplementary Figure 17 Composite time series of the running 3-month mean SST anomaly for the Niño 3.4 region (5°N–5°S, 120°–170°W) (N3.4, an ENSO index, °C) for observations and predictions for El Niño–Southern Oscillation events in the hindcasting period (2002–2016). a, Composite time series of N3.4 for all El Niño events. Blue dashed line is the average prediction by statistical models, red for the ACE+N3.4 model. Black solid line is observations. Bars indicate the amplitude of models' relative advantage ($\text{relative advantage}_{\text{model A}\rightarrow\text{model B}} = |\text{Model A}-\text{Observation}| - |\text{Model B}-\text{Observation}|$) between the average of the dynamical models and the ACE+N3.4 model. Blue (orange) bars represent the advantage of the statistical models average relative to the ACE+N3.4 model (ACE+N3.4 model relative to the statistical model average). DJF-1 and DJF0

represent the December–February in last year and the year concurring with El Niño, respectively. **b**, Same as **a**, but for La Niña.

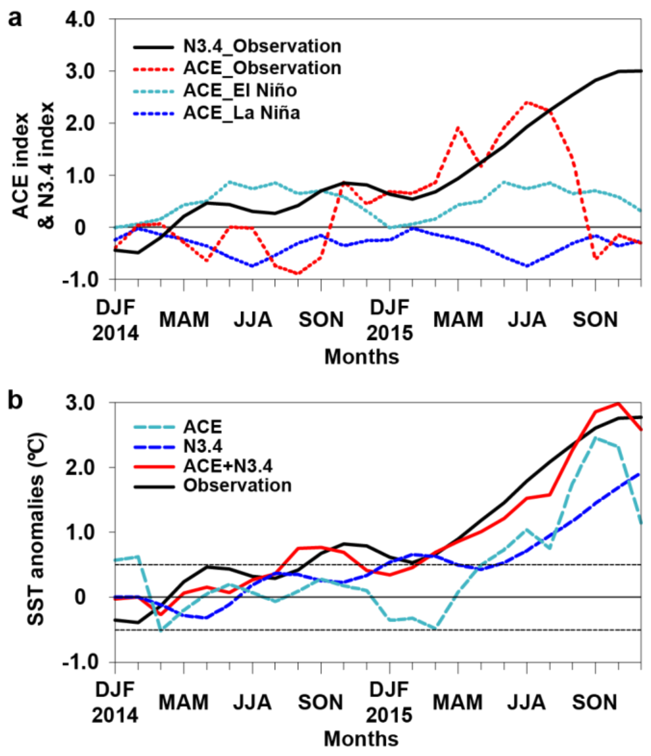

Supplementary Figure 18 Time series of the running 3-month mean SST anomaly for the Niño 3.4 region (5°N–5°S, 120°–170°W) (N3.4, an ENSO index, 2014–2015) for observations and predictions, as well as the observed accumulated cyclone energy (ACE) anomalies over the western North Pacific (10°–20°N, 135°–170°E). **a**, Standardized time series of the observed N3.4 and ACE anomalies. Black solid line is the observed N3.4; red dotted line is the observed ACE; turquoise (blue) line is the composite of ACE anomalies during El Niño (La Niña). **b**, Time series of average N3.4 (°C) from 2014 to 2015. Blue dashed line is the prediction from N3.4 model, turquoise for ACE model; red solid line for ACE+N3.4 model, black for observations.

Supplementary Tables

Supplementary Table 1 Explained percentages (%, red) of the running 3-month mean SST anomaly for the Niño 3.4 region (5°N–5°S, 120°–170°W) (N3.4, an ENSO index) from July to December by the preceding (3 months earlier) accumulated cyclone energy (ACE) anomalies over the western North Pacific (10°–20°N, 135°–170°E) and the corresponding accumulated cyclone energy anomalies ($m^2 s^{-2}$, black) 3 months earlier during El Niño developing years for the period 1970–2016. An El Niño developing year is defined when the above-moderate El Niño events (including moderate events) develops from weak to strong.

		4(7)	5(8)	6(9)	7(10)	8(11)	9(12)
1972	ACE	-39.70	28.86	509.23	577.85	554.46	294.35
	Percentage	-23.86	31.26	190.30	170.76	138.24	71.71
1982	ACE	-14.55	2.32	123.91	229.69	207.30	179.06
	Percentage	7.65	17.34	65.77	99.59	70.78	47.50
1986	ACE	105.50	109.52	132.00	-2.73	48.13	24.62
	Percentage	-93.01	-134.86	-286.49	10.95	49.87	26.26
1991	ACE	64.76	41.99	17.72	-94.13	30.03	166.27
	Percentage	65.66	36.98	22.75	-13.10	26.77	73.89
1994	ACE	-21.19	-28.57	-55.19	-45.68	108.09	311.33
	Percentage	2.25	1.13	-13.07	-5.76	67.83	134.56
1997	ACE	163.00	250.10	65.90	186.24	93.17	261.34
	Percentage	133.73	94.60	29.60	45.07	28.60	46.96
2002	ACE	27.79	10.72	368.66	347.45	330.06	-77.17
	Percentage	41.25	21.39	137.64	111.31	99.74	-0.81
2009	ACE	-34.54	-40.47	-77.14	-43.39	79.24	310.50
	Percentage	74.90	-34.49	-24.80	-1.51	45.46	115.77
2015	ACE	242.62	172.30	408.54	567.04	538.22	266.81
	Percentage	88.68	55.93	92.96	103.11	86.48	47.03

Note: The numbers 4–12 in the first row represent April–December, respectively.
